# Abnormal Kinetochore-Generated Pulling Forces from Expressing a N-Terminally Modified Hec1

**DOI:** 10.1371/journal.pone.0016307

**Published:** 2011-01-28

**Authors:** Marta Mattiuzzo, Giulia Vargiu, Pierangela Totta, Mario Fiore, Claudio Ciferri, Andrea Musacchio, Francesca Degrassi

**Affiliations:** 1 Institute of Molecular Biology and Pathology, National Research Council of Italy, Rome, Italy; 2 Department of Experimental Oncology, European Institute of Oncology, Milan, Italy; 3 Department of Molecular and Cell Biology, University of California, Berkeley, California, United States of America; University of Edinburgh, United Kingdom

## Abstract

**Background:**

Highly Expressed in Cancer protein 1 (Hec1) is a constituent of the Ndc80 complex, a kinetochore component that has been shown to have a fundamental role in stable kinetochore-microtubule attachment, chromosome alignment and spindle checkpoint activation at mitosis. *HEC1* RNA is found up-regulated in several cancer cells, suggesting a role for *HEC1* deregulation in cancer. In light of this, we have investigated the consequences of experimentally-driven Hec1 expression on mitosis and chromosome segregation in an inducible expression system from human cells.

**Methodology/Principal Findings:**

Overexpression of Hec1 could never be obtained in HeLa clones inducibly expressing C-terminally tagged Hec1 or untagged Hec1, suggesting that Hec1 cellular levels are tightly controlled. On the contrary, a chimeric protein with an EGFP tag fused to the Hec1 N-terminus accumulated in cells and disrupted mitotic division. EGFP- Hec1 cells underwent altered chromosome segregation within multipolar spindles that originated from centriole splitting. We found that EGFP-Hec1 assembled a mutant Ndc80 complex that was unable to rescue the mitotic phenotypes of Hec1 depletion. Kinetochores harboring EGFP-Hec1 formed persisting lateral microtubule-kinetochore interactions that recruited the plus-end depolymerase MCAK and the microtubule stabilizing protein HURP on K-fibers. In these conditions the plus-end kinesin CENP-E was preferentially retained at kinetochores. RNAi-mediated CENP-E depletion further demonstrated that CENP-E function was required for multipolar spindle formation in EGFP-Hec1 expressing cells.

**Conclusions/Significance:**

Our study suggests that modifications on Hec1 N-terminal tail can alter kinetochore-microtubule attachment stability and influence Ndc80 complex function independently from the intracellular levels of the protein. N-terminally modified Hec1 promotes spindle pole fragmentation by CENP-E-mediated plus-end directed kinetochore pulling forces that disrupt the fine balance of kinetochore- and centrosome-associated forces regulating spindle bipolarity. Overall, our findings support a model in which centrosome integrity is influenced by the pathways regulating kinetochore-microtubule attachment stability.

## Introduction

The kinetochore (KT) is the protein complex responsible for mediating attachment of sister chromatids to the mitotic spindle and for directing chromosome movements during mitosis. It is also the chromosomal site that generates the signal preventing anaphase onset in the presence of incorrect attachment or no attachment to spindle microtubules (MTs) [1], [2]. Thus, the KT is at the hearth of the spindle checkpoint, the signaling pathway ensuring an equal distribution of the genetic material at mitosis [3], [4]. Consistent with these multiple functions, kinetochore malfunctioning results in chromosome segregation errors during mitosis and produce aneuploidy [5], a condition that was recognized already one hundred years ago as an ubiquitous feature of human tumour cells [6]. Nowadays, numerous lines of evidence provide strong support for a crucial role of altered chromosome numbers in the initiation and/or progression of cancer [4], [7]–[9]. Consistently, nearly all solid tumours exhibit chromosome instability (CIN), *i.e.* an increased rate of chromosome mis-distribution at mitosis [10], a feature which may greatly contribute to the plasticity of the cancer genome and to acquired chemoresistance [4]. Compelling evidence shows that genetic or epigenetic alterations of spindle checkpoint signaling proteins promote chromosome segregation errors, aneuploidy and polyploidy in cultured mammalian cells and in experimental organisms [4], [9] and expression of these factors is often deregulated in cancer samples [4], [11]. At opposite, investigations on cancer-related genetic or epigenetic alterations in KT structural proteins or protein mediating kinetochore-microtubule (KT-MT) attachment are still scanty [11]–[13].

Highly Expressed in Cancer protein 1 (Hec1) [14] is a constituent of the evolutionary conserved Ndc80 KT complex which is formed by the Hec1 (Ndc80 in yeast), Nuf2, Spc24 and Spc25 subunits. The globular N-terminal heads of Hec1 and Nuf2 and the globular C-terminal heads of Spc24 and Spc25 are located at the opposite ends of a central rod domain, forming a dumb-bell shaped 50 nm long complex [15], [16]. The complex localizes to the outer KT plate, where MT plus-ends terminate [17], and is required for robust KT-MT attachment and localization of regulatory proteins to the outer KT [18]–[24]. Consistently, RNAi-mediated Hec1 depletion leads to defective mitotic checkpoint signaling, abnormal mitotic exit and apoptosis [18], [19], [22], [25]. *In vitro* interaction studies with purified Ndc80 complexes have shown that the Hec1-Nuf2 head binds directly the MT lattice [26]–[28], leading to the conclusion that the Ndc80 complex directly connects MTs to the KT in vertebrate cells [24], [26]–[28]. In line with its role at mitosis, Hec1 is abundantly produced in rapidly dividing tissues and its expression is cell cycle regulated, peaking at mitosis [14], [29]. Genome-wide expression profile analysis demonstrates that *HEC1* is up-regulated in brain, liver, breast and lung tumor cells [11], [30]–[32] and in several cancer cell lines [14], [29], [31], [32]. Moreover, Hec1 overexpression has been associated to poor clinical prognosis in non small cell lung cancers, breast cancers and patients with multiple cancers [33], [34].

The crucial role of the Ndc80 complex in mediating a key function for chromosome segregation in mitosis and the recurrent *HEC1* up-regulation in different human cancers suggest that *HEC1* deregulation may be an important step in the multistage process of cancer. Indeed, Hec1 overexpression in an inducible mouse model has been shown to result in mitotic checkpoint hyperactivation in MEFs and tumor formation in different tissues [35]. Here, we investigated the consequences of experimentally driven Hec1 deregulation on mitosis and chromosome segregation in an inducible expression system from human cells in which the transgene is inserted at a single chromosomal site [36]. Overexpression of Hec1 could never be obtained in inducible HeLa clones expressing C-terminally tagged Hec1 (Hec1-EGFP) or untagged Hec1, suggesting that Hec1 cellular levels are tightly controlled. On the contrary, inducible or transient expression of a Hec1 fusion protein with an EGFP tag at its N-terminus (EGFP-Hec1) disrupted mitotic division by producing altered chromosome segregation within multipolar spindles. We show here that expression of EGFP N-terminally tagged Hec1 impairs mitosis by perturbing KT-MT attachment dynamics and KT-associated motor protein function.

## Results

### N-terminally tagged Hec1 assembles a mutant Ndc80 complex

**Figure 1:**
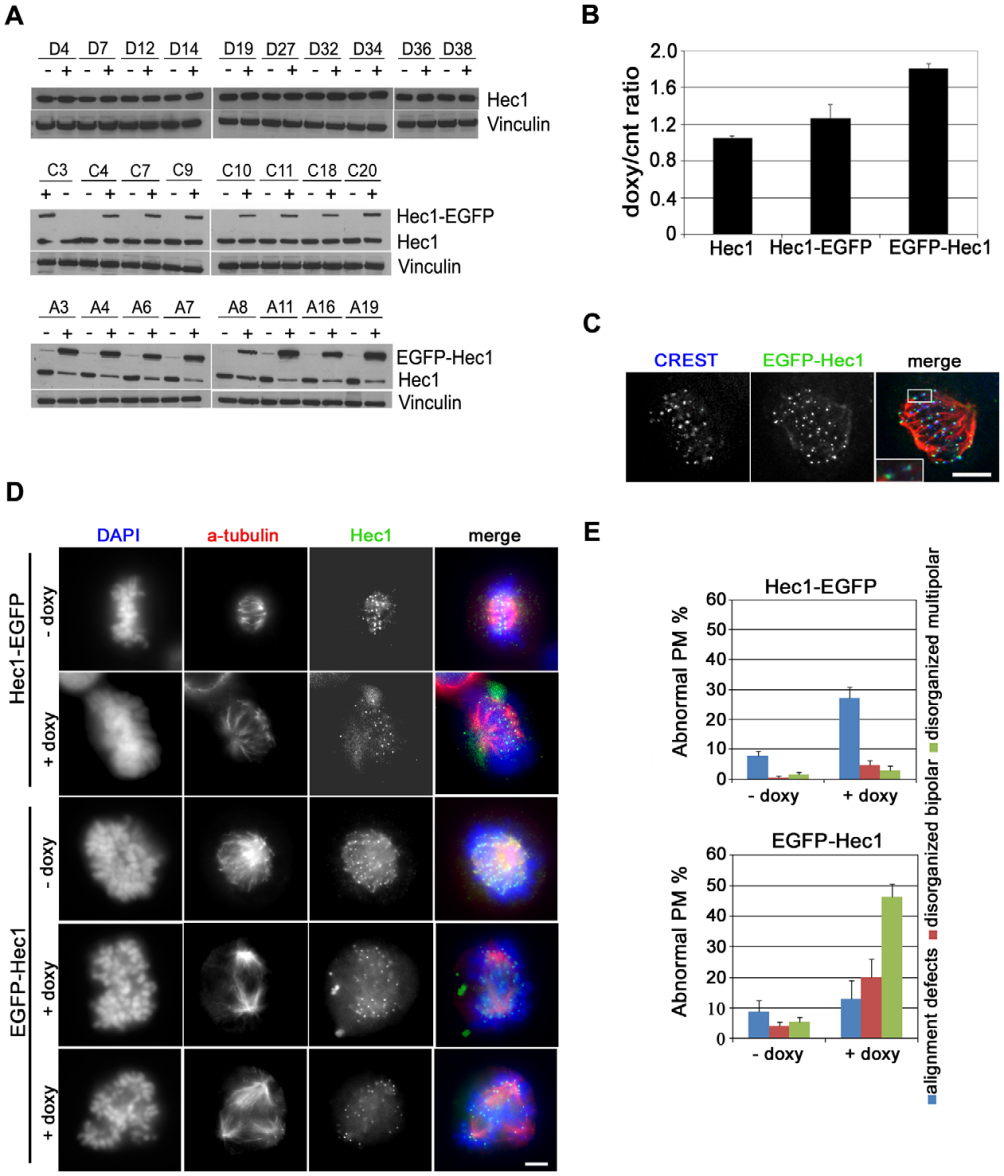
Inducible Hec1 expression in HeLa clones. (A) Western blot analysis using an Hec1 antibody before (-) and after (+) doxycycline addition in HeLa clones expressing Hec1 (D clones), Hec1-EGFP (C clones) or EGFP-Hec1 (A clones). (B) Densitometric analysis of Hec1 expression in the different clones as measured by the ratio of total Hec1 (endogenous + exogenous) in induced (doxy) *vs* uninduced (cnt) conditions after normalization for vinculin expression. Data are mean ± SE of the ratios obtained in clones expressing untagged Hec1 (n = 10), Hec1-EGFP (n = 8) or EGFP-Hec1 (n = 8). (C) EGFP-Hec1 (green) localization after doxy induction in a prometaphase cell stained for CREST (blue) and tubulin (red). The boxed 2-fold enlargement shows EGFP-Hec1 localization with respect to the inner kinetochore marker CREST. Bar, 5 µm. (D) Chromosome congression and spindle organization before (-doxy) and after (+doxy) induction of Hec1-EGFP or EGFP-Hec1 expression as shown by α-tubulin (red) and DAPI (blue) staining. In uninduced samples Hec1 is visualized by antibody staining (green). Hec1-EGFP/-doxy: normal metaphase; Hec1-EGFP/+doxy: late prometaphase with unaligned chromosomes; EGFP-Hec1/-doxy: normal prometaphase; EGFP-Hec1/+doxy (fourth row): disorganized bipolar prometaphase; EGFP-Hec1/+doxy (last row): disorganized multipolar prometaphase. Bar, 5 µm. (E) Quantitative analysis of prometaphase abnormalities in Hec1-EGFP or EGFP-Hec1 expressing clones. Data are mean ± SE of 300 prometaphases (PM) for each condition in three independent experiments. Alignment defects were significantly higher in Hec1-EGFP expressing mitoses than in uninduced cells (P<0.01, t-test); disorganized bipolar prometaphases (P<0.05, t-test) and disorganized multipolar prometaphases (P<0.01, t-test) were significantly higher in EGFP-Hec1 expressing mitoses than in uninduced mitoses.

To investigate the role of Hec1 in generating chromosome instability in human cells, inducible vectors expressing wild type untagged Hec1 (Hec1) or Hec1 fused to the enhanced green fluorescent protein at the N-terminus (EGFP-Hec1) or C-terminus (Hec1-EGFP) of the protein were constructed. Several stable HeLa clones were generated by Flp recombinase-mediated integration of these doxycycline-responsive cassettes into a single pre-defined site in the Flp-In HeLa cells, a strategy that eliminates differences in expression levels that may arise because of integration at different chromosomal sites [36]. Isolated clones were subjected to doxycycline (doxy) treatment to induce expression of the differently modified Hec1 proteins (Figure 1A). Surprisingly, western blot analysis showed that Hec1 intracellular levels remained unchanged when transcription of untagged Hec1 was activated by exposure to doxy (Figure 1A, upper row). In clones expressing Hec1 fused at its C-terminus with EGFP, the exogenous protein (Hec1-EGFP) was expressed at levels lower than endogenous Hec1 (Figure 1A, middle row). Accordingly, western blot densitometry demonstrated that total intracellular Hec1 content (Hec1+Hec1-EGFP) in Hec1-EGFP expressing cells was only 20% higher than Hec1 content in uninduced conditions (Figure 1B). This low expression of C-terminally tagged Hec1 was confirmed after a wide range of doxy concentrations in a representative clone (Figure S1A). On the other hand, EGFP N-terminally tagged-Hec1 (EGFP-Hec1) accumulated quite heavily after induction. Concomitantly, endogenous protein content was dramatically reduced so that the exogenous protein constituted the major fraction of Hec1 in EGFP-Hec1 expressing cells (Figure 1A, bottom row and Figure S1B). Furthermore, the mean content of total Hec1 (Hec1+EGFP-Hec1) in eight EGFP-Hec1 expressing clones was 72% higher than endogenous Hec1 in uninduced conditions (Figure 1B). These observations indicate that Hec1 intracellular content is tightly regulated, so that exogenously expressed Hec1 is transcribed at low efficiency or promptly degraded when untagged Hec1 or Hec1-EGFP are expressed. On the contrary, exogenous EGFP-Hec1 is preferentially retained in EGFP-Hec1 expressing clones at the expense of the endogenous protein.

Next, we analyzed the mitotic process in uninduced or induced conditions in two clones that were chosen as representative of Hec1-EGFP or EGFP-Hec1 expressing cells, *i.e.* C7 and A6 clones, respectively (see Figure 1A). Microscopic inspection demonstrated that mitoses expressing Hec1-EGFP or EGFP-Hec1 harbored the chimeric protein at the KT, with a localization external to the CREST signal, a marker for the inner KT (Figure 1C, see inset), and in close association with the antibody signal for the Spc24/Spc25 subunits of the Ndc80 complex (Figure S2). These localization studies showed that exogenously expressed Hec1 was correctly recruited at the outer KT within a chimeric Ndc80 complex. Combined analysis of chromosome organization and spindle structure by DNA staining and α-tubulin immunostaining demonstrated that expression of Hec1-EGFP only slightly modified the mitotic process (Figure 1D). Indeed, no overt mitotic phenotypes were observed except for an induction of chromosome alignment defects, *i.e.* late prometaphase cells in which few KTs localized external to the metaphase plate (Figure 1D, second row). On the contrary, EGFP-Hec1 cultures exhibited severe defects in prometaphase chromosome congression as shown by an increase in mitotic index (not shown) and a sharp decrease in the percentage of metaphases after doxy induction (4.2±2.1 *vs* 22.7±3.7). Most EGFP-Hec1 expressing prometaphases were disorganized prometaphases, *i.e*. prometaphases in which highly condensed chromosomes were abnormally clustered (Figure 1D, EGFP-Hec1/+doxy) as compared with the homogenous distribution of the chromosomes within a normal early prometaphase (Figure 1D, EGFP-Hec1/-doxy). Furthermore, α-tubulin immunostaining demonstrated the presence of both disorganized prometaphases with a bipolar spindle (Figure 1D, fourth row) and disorganized prometaphases harboring a multipolar spindle (Figure 1D, last row). Altogether, more than 80% EGFP-Hec1 prometaphases were abnormal and disorganized multipolar prometaphases were the predominant phenotype (Figure 1E, lower graph). A dramatic reduction in the frequency of ana-telophases was also found together with a high frequency of segregation abnormalities in the few anaphases recovered (not shown). Similar mitotic defects were observed in HeLa cells transiently transfected with an EGFP-Hec1 construct, indicating that expression of the same chimeric protein through inducible expression or transient transfection produced the same mitotic alterations (Figure S3A, B and C). Consistent with the severe mitotic phenotypes observed, live cell imaging showed that EGFP-Hec1 cells experienced a significant lengthening of the time spent in mitosis with a dramatic prolongation of the cytokinesis time (Figure S3D). This was accompanied by the complete failure of cytokinesis in all recorded cells, due to an aberrant chromosome segregation in which chromatin was laying at the equator of the cell during telophase (Figure S3E). Moreover, transient expression of EGFP-Hec1 in other mammalian cell lines, such as osteosarcoma (U2OS) or diploid colon cancer (HCT116) cells generated the same phenotypes, suggesting that mitotic disruption brought about by the presence of the chimeric protein was a general phenomenon (Figure S4).

**Figure 2:**
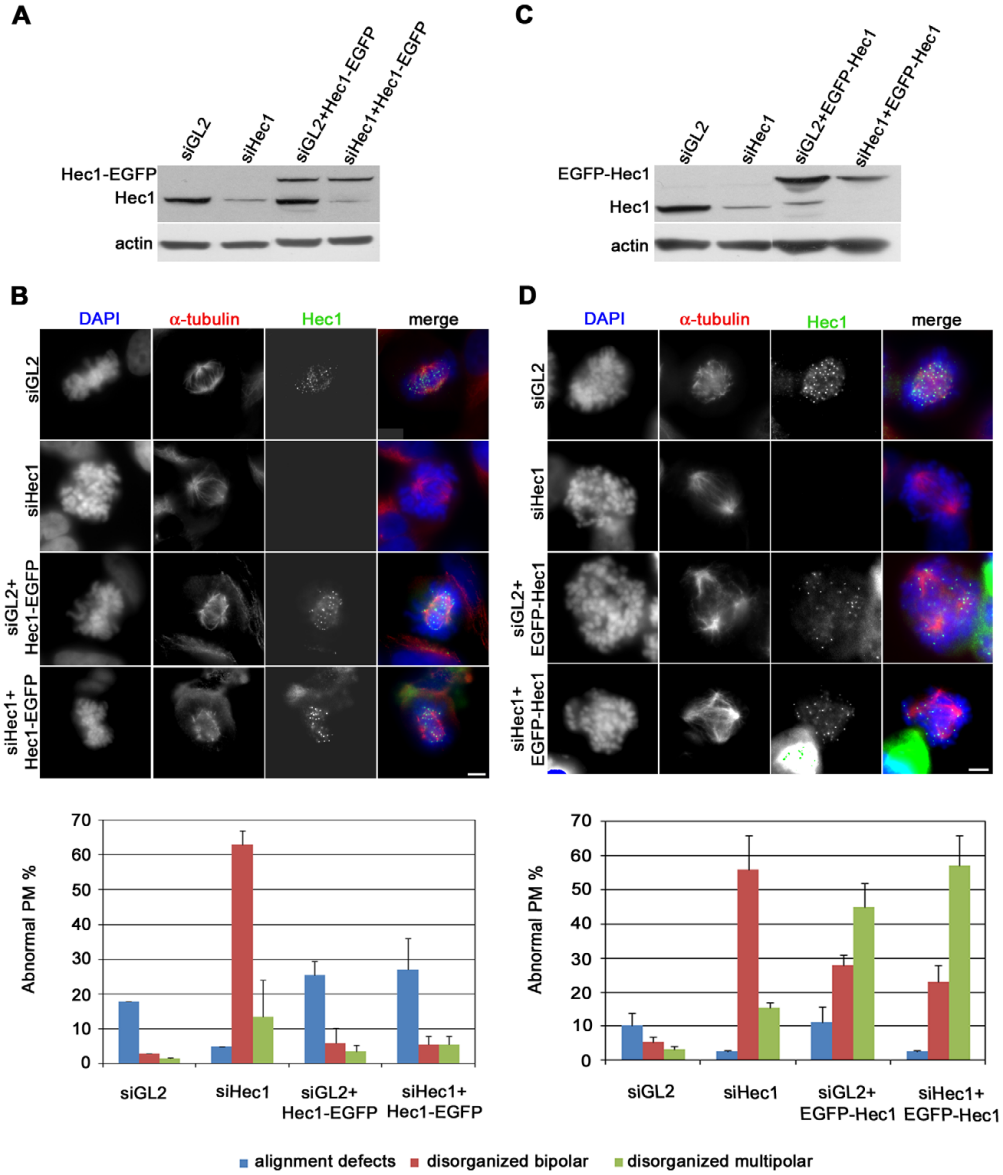
Expression of EGFP-Hec1 does not rescue the mitotic phenotypes induced by Hec1 depletion. (A) Western blot analysis of Hec1 depletion with or without induced Hec1-EGFP expression. (B) Chromosome congression and spindle organization after α-tubulin (red) and DAPI staining (blue) in control (siGL2) or Hec1 silenced (siHec1) mitoses or mitoses expressing Hec1-EGFP after control silencing (siGL2+ Hec1-EGFP) or after Hec1 silencing (siHec1+ Hec1-EGFP). In siGL2 and siHec1 samples, Hec1 is visualized by antibody staining. The graph reports the frequencies of prometaphase abnormalities in the different conditions in Hec1-EGFP cells. Data are mean ± SE of 200 PM scored for each condition in two independent experiments. (C) Western blot analysis of Hec1 depletion with or without induced EGFP-Hec1 expression. (D) Chromosome congression and spindle organization after α-tubulin (red) and DAPI staining (blue) in control (GL2) or Hec1 silenced (siHec1) mitoses or mitoses expressing EGFP-Hec1 after control silencing (siGL2+ EGFP-Hec1) or after Hec1 silencing (siHec1+ EGFP-Hec1). In siGL2 and siHec1 samples, Hec1 is visualized by antibody staining. The graph reports the frequencies of prometaphase abnormalities in the different conditions in EGFP-Hec1 cells. Data are mean ± SE of 200-300 PM for each condition in two-four experiments. Disorganized bipolar prometaphases were significantly induced by siHec1 in both clones (P<0.01, t-test) and disorganized multipolar prometaphases were significantly induced after siHec1 in EGFP-Hec1 cells (P<0.05, t-test).

To better understand the dependence of the mitotic phenotypes on tag localization, we directly assessed the functionality of N-terminally or C-terminally tagged Hec1 by analyzing the capacity of these two proteins to rescue the mitotic phenotypes induced by siRNA-mediated depletion of endogenous Hec1. Hec1 was depleted in exponentially growing cells from Hec1-EGFP or EGFP-Hec1 clones and expression of the tagged protein was successively induced by doxy addition 24 h before harvesting. In these conditions, endogenous Hec1 was depleted by more than 70% in both clones after siRNA transfection (Figure 2A and C). Hec1 partial depletion caused a typical spindle checkpoint-mediated prometaphase arrest characterized by increased mitotic index (not shown) and highly condensed chromosomes not interacting with spindle MTs (Figure 2B and D, siHec1) [18]. When Hec1-EGFP expression was induced the checkpoint phenotype was abolished so that >60% of mitoses were normal prometaphases and metaphases. Remaining prometaphases displayed alignment defects, indicative of a rescue from the lack of KT-MT interaction (Figure 2B, graph). When EGFP-Hec1 expression was induced in the depleted background, the types and frequencies of mitotic anomalies were almost identical to the ones observed in non-silenced conditions (Figure 2D, graph), showing that abnormal mitotic progression and spindle abnormalities occurred independently of Hec1 protein levels but that the presence of EGFP-Hec1-containing Ndc80 complexes was sufficient to drive mitotic failure. These findings demonstrate that congression defects and induction of multipolar spindles in EGFP-Hec1 expressing mitoses are independent from intracellular Hec1 levels but their occurrence is linked to the expression of the N-terminally tagged Hec1 assembled into the Ndc80 complex.

### EGFP-Hec1 expression induces centrosome fragmentation mediated by spindle forces

**Figure 3:**
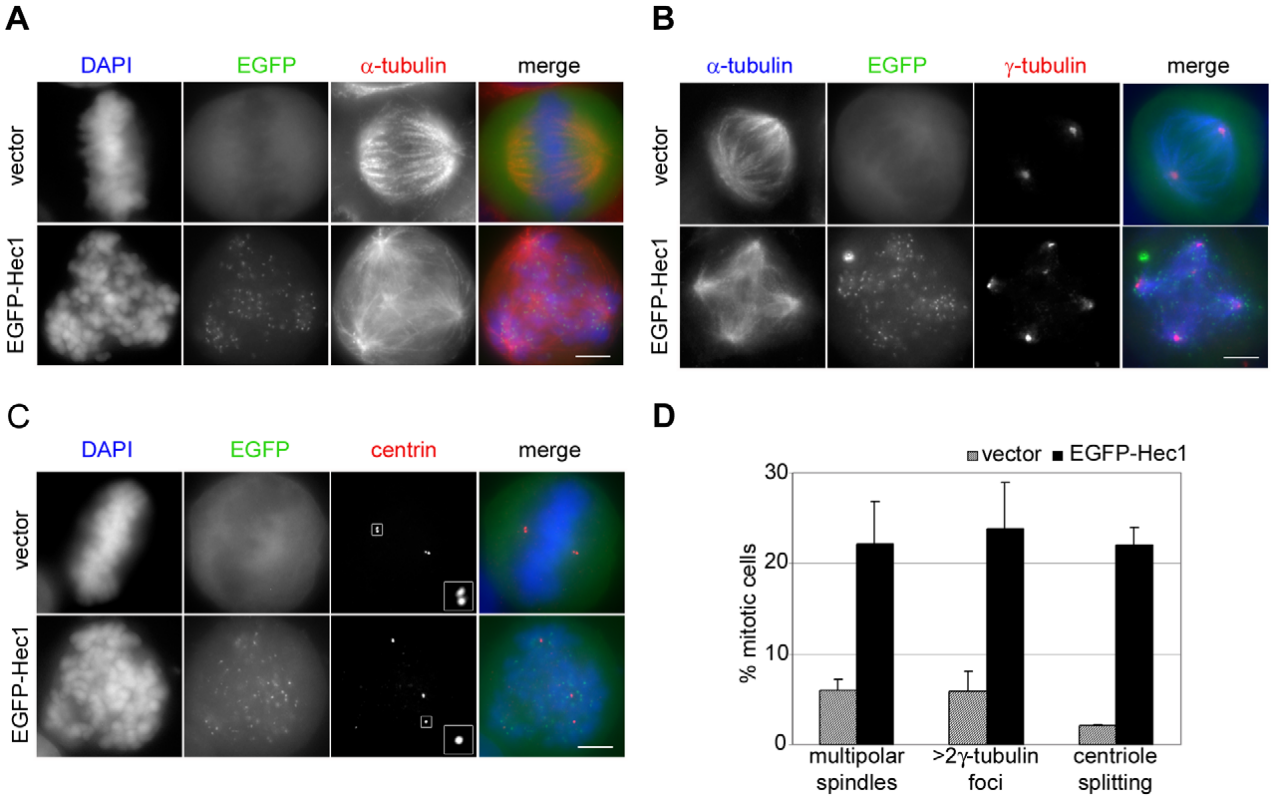
EGFP-Hec1 expression induces multipolar spindles and centrosomal abnormalities. (A) Vector or EGFP-Hec1 (green) transfected cells stained for α-tubulin (red) and DAPI (blue). The EGFP-Hec1 disorganized prometaphase exhibited a multipolar spindle. (B) Vector or EGFP-Hec1 (green) transfected cells stained with antibodies to α-tubulin (blue) and γ-tubulin (red). 5 γ-tubulin spots connected to spindle microtubules were present in the EGFP-Hec1 cell. (C) Vector or EGFP-Hec1 (green) transfected cells stained with antibodies to centrin (red). The boxed 3-fold enlargements show a centriole pair in the vector-transfected metaphase and one single centriole in the EGFP-Hec1 disorganized prometaphase. (D) Mean percentage (± SE) of multipolar spindles detected by α-tubulin staining (n = 300 from triplicate assays), >2 γ-tubulin foci (n>200 from triplicate assays) and centriole splitting (n = 200 from duplicate assays) in vector and EGFP-Hec1 transfected mitoses. Multipolar spindles (α-and γ-tubulin staining, P<0.05, t-test) and splitted centrioles (P<0.01, t-test) were significantly higher in EGFP-Hec1 expressing mitoses than in vector cells. Bars, 5 µm.

To identify the cause of this impairing effect of N-tagged Hec1 on Ndc80 complex function, we accurately analyzed spindle pole organization in EGFP-Hec1 transiently transfected HeLa cells. As described above, a large fraction of EGFP-Hec1 mitoses possessed a multipolar spindle with well developed asters associated with MT bundles (Figure 3A). Double immunostaining with anti α-tubulin and anti γ-tubulin antibodies showed that each MT aster was linked to a γ-tubulin focus (Figure 3B) demonstrating that more spindle poles were present in EGFP-Hec1 cells. To assess whether supernumerary spindle poles originated from centrosomal fragmentation, we analyzed the core of the centrosomes, *i.e*. the centrioles, by immunostaining centrin, the major centriole component. In a normal mitosis each spindle pole harbored two paired centrioles, visible as two adjacent centrin dots (Figure 3C, vector). In EGFP-Hec1 multipolar prometaphases, each spindle pole contained one single centrin dot, indicative of an abnormal separation of the paired centrioles in both centrosomes (Figure 3C, EGFP-Hec1). These data show that EGFP-Hec1 expression induces the appearance of supernumerary MT foci caused by centriole splitting. Moreover, we did not observe an increase in the percentage of first post-transfection mitoses showing more than 4 centrin spots (8.4±3.0 in vector *vs* 9.5±1.3 in EGFP-Hec1). However, in EGFP-Hec1 multipolar prometaphases each spindle pole contained a single centriole, because paired centrioles had splitted. These findings demonstrate that spindle multipolarity in EGFP-Hec1 cells does not originate from centrosome overduplication intervening in the preceding interphase but it is likely to be generated during mitosis. Finally, frequencies of multipolar prometaphases in EGFP-Hec1 cells (as detected by α-tubulin immunostaining) were almost equal to the frequencies of prometaphases with supernumerary γ-tubulin foci or single centrioles in spindle poles (Figure 3D), demonstrating that centriole splitting was the cause for pole fragmentation in these cells.

**Figure 4:**
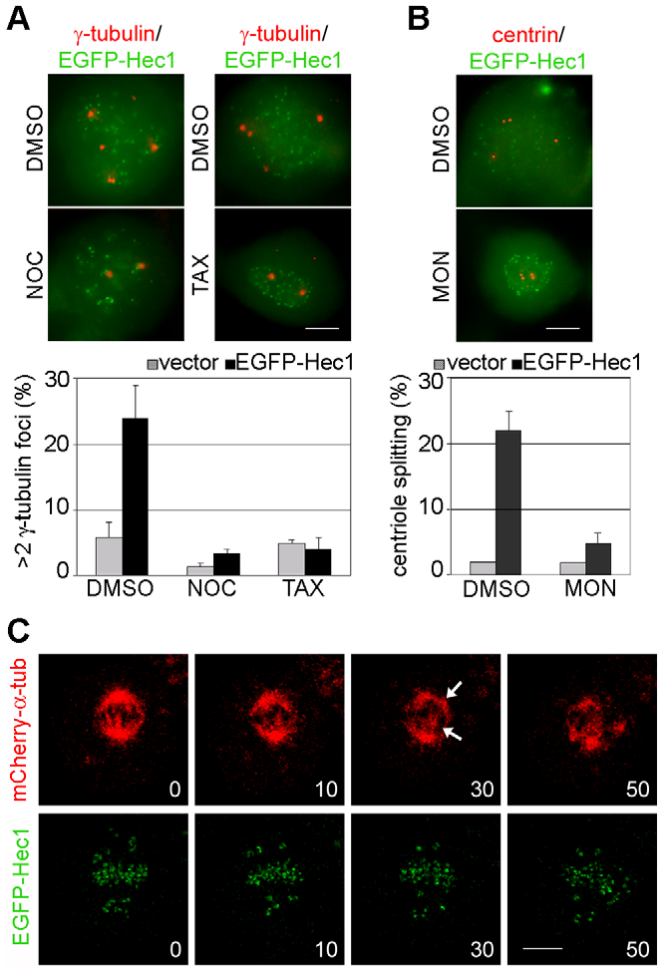
Spindle pole fragmentation is due to an unbalance of forces within the mitotic spindle. (A) Merged image of EGFP-Hec1 (green) expressing prometaphases stained for γ-tubulin (red) after incubation with nocodazole (NOC), taxol (TAX) or DMSO for the last 4 h expression time. Only 2 γ-tubulin signals are present after NOC or TAX treatment in the EGFP-Hec1 cells. The graph shows the mean percentage (± SE) of mitotic cells with >2 γ-tubulin foci in vector or EGFP-Hec1 cells treated with NOC, TAX or DMSO (n = 200 from duplicate assays). (B) Merged image of EGFP-Hec1 (green) expressing prometaphases stained for centrin (red) after incubation with monastrol (MON) or DMSO for the last 4 h expression time. The graph shows the mean percentage (± SE) of mitotic cells with splitted centrioles in vector or EGFP-Hec1 cells treated with MON or DMSO (n = 200 from duplicated assays). (C) HeLa cells cotransfected with EGFP-Hec1 (green) and mCherry-α-tubulin (red) vectors were recorded by time-lapse microscopy during mitosis. mCherry- α-tubulin shows that the late prometaphase showing many unaligned KTs initially possessed a bipolar spindle (0). KTs attempted to congress within the bipolar spindle (10), but, then, two supernumerary spindle poles appeared under mCherry-tubulin fluorescence (30, arrows). Spindle poles successively moved apart and the KTs re-organized within the multipolar spindle (50). Time is given in min. Bars, 5 µm.

Spindle bipolarity is established and maintained during prometaphase/metaphase through a finely tuned balance of MT-mediated forces exerted on different locations (centrosomes, KTs, chromosome arms) by minus-end directed dyneins and plus-end directed kinesins [37], [38]. We hypothesized that an unbalance of forces, generating an excessive pulling force on centrosomes was at the origin of centriole splitting in EGFP-Hec1 prometaphases. To test this hypothesis, we depolymerized spindle MTs by adding the MT inhibitor nocodazole (NOC) 44 h post-transfection, fixed cells 4 h later and monitored centrosomal fragmentation. The increase in γ-tubulin foci observed in EGFP-Hec1 cells was abolished when cells entered mitosis in the presence of NOC (Figure 4A, NOC). Addition of taxol (TAX), a spindle poison responsible for MT stabilization at low doses, also rescued centrosome fragmentation in EGFP-Hec1 expressing cells (Figure 4A, TAX), suggesting that dynamic MTs are required for centrosomes to fragment. To investigate the origin of centriole splitting, we used monastrol (MON), a specific inhibitor of Eg5, the kinesin responsible for the separation of centrosomes at M-phase entry [39]. More than 90% of MON-treated EGFP-Hec1 prometaphases showed two pairs of adjacent centrioles at the centre of a monopolar spindle (Figure 4B), indicating that centriole splitting intervenes after centrosome separation at prophase. Hence, these data demonstrate that spindle pole fragmentation in EGFP-Hec1 expressing cells is a MT-dependent mitotic process and suggest that centrosome disruption and centriole splitting are due to an unbalance of the forces generated on the spindle.

To corroborate this hypothesis, we further examined spindle dynamics of EGFP-Hec1 mitosis using live imaging of cells expressing a mCherry-tagged version of α-tubulin [40]. Visualization of spindle MTs *in vivo* unambiguously demonstrated that multipolar spindles induced by EGFP-Hec1 expression arise from bipolar spindles during prometaphase. Indeed, the prometaphase in Figure 4C showed a bipolar spindle and many unaligned KTs at the beginning of the observation. While the chromosomes attempted to congress within a bipolar spindle, two supernumerary spindle poles appeared under the mCherry-tubulin fluorescence (30 min); thereafter, the four spindle poles further separated and the chromosomes re-organized within a multipolar spindle (50 min). Formation of a multipolar spindle was always observed from a bipolar one in EGFP-Hec1/mCherry-tubulin mitoses (n = 10). Taken together, these results demonstrate that forces generated while chromosomes attempt to congress to the spindle equator, namely pulling forces exerted on centrosomes trigger spindle pole fragmentation.

### EGFP-Hec1 produces persisting kinetochore-microtubule mis-attachments

Given the fundamental role of Hec1 in MT interaction at KT we decided to investigate the nature of KT-MT attachments in EGFP-Hec1 expressing cells. To this aim, we treated cells with the proteasome inhibitor MG132 to delay anaphase onset and allow longer time for correction of erroneous KT-MT attachments [41]. Under these conditions, congression defects were only partially corrected in EGFP-Hec1 cells when anaphase was delayed for 2 h (35% of congression defects in EGFP-Hec1+MG132 *vs* 53% in EGFP-Hec1 n≥100), implying a defective correction of EGFP-Hec1-induced KT-MT interactions.

**Figure 5:**
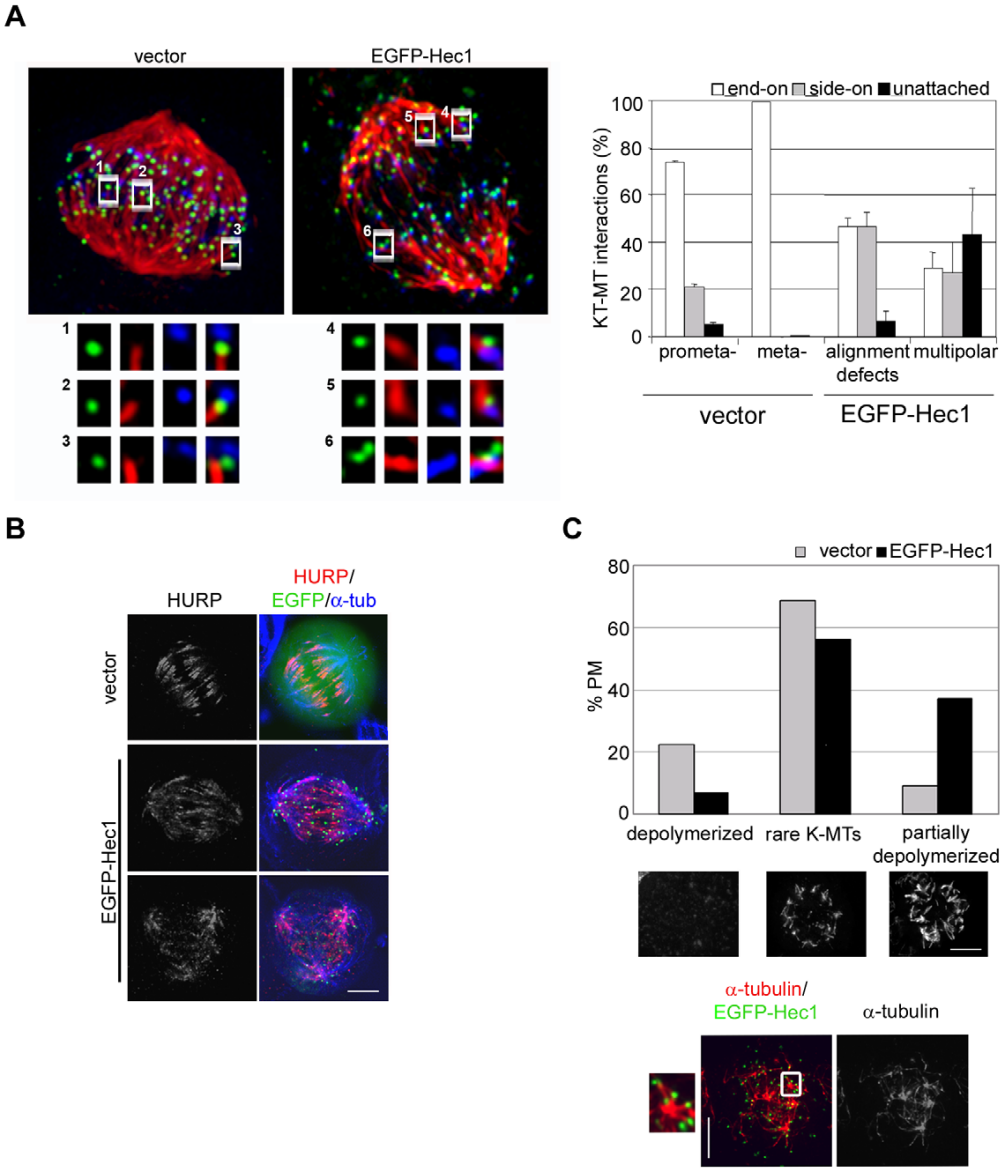
Erroneous kinetochore-microtubule attachments in EGFP-Hec1 cells stabilize K-fibers. (A) KT-MT attachments in a late prometaphase (vector) and an EGFP-Hec1 expressing disorganized bipolar prometaphase (EGFP-Hec1) exposed to MG132 for 2 h and stained for CREST (blue) and α-tubulin (red) after calcium buffer treatment. Kinetochores (green) are visualized by anti-Hec1 antibody (vector) or EGFP-Hec1 (EGFP-Hec1). Maximum projections from deconvolved z-stacks are shown. Insets show a 3-fold enlargement of 2-3 slices from the boxed regions. In the vector prometaphase two end-on attachments (1,2) and one side-on attachment close to the MT end (3) are shown. In the EGFP-Hec1 cell two side-on attachments similar to the one observed in the vector cell (4,5) and one side-on attachment along a continuing MT (6) are reported. Bar 5 µm. The graph reports a quantitative analysis of the different types of attachments on 175–284 kinetochores in 2–3 cells for each mitotic stage. Alignment defects include disorganized bipolar prometaphases. (B) HURP (red) and α-tubulin (blue) staining in vector or EGFP-Hec1 cells. HURP localization extended towards the MT minus-ends in the EGFP-Hec1 cell. (C) Quantitative analysis of MT depolymerization in NOC-treated vector and EGFP-Hec1 prometaphases (PM) (n = 200). Representative examples of the different classes of MT depolymerization are presented below the graph (α-tubulin staining). Localization of residual MTs (α-tubulin, red) with respect to KTs (EGFP-Hec1, green) in a EGFP-Hec1 expressing prometaphase after NOC treatment. Inset shows a 3-fold enlargement of the boxed region in the merge.

To visualize KT-MT attachments we depolymerized unstable MTs by calcium treatment [42] in MG132 exposed cells, and simultaneously visualized centromeres, KTs and MTs to identify end-on (Figure 5A, insets 1,2) and side on (insets 3-6) attachments in vector and EGFP-Hec1 transfected cells. A quantitative analysis of KTs with calcium-stable KT fibres (Figure 5A, graph) demonstrated that EGFP-Hec1 expression led to a clear increase in calcium-resistant side-on attachments in bipolar prometaphases with alignment defects (46.4±6.2% compared with 21±1.3% in vector prometaphase cells), indicating a longer permanence of these interactions during prometaphase in EGFP-Hec1 mitoses. Side-on interactions and unattached KTs were also largely present in multipolar EGFP-Hec1 disorganized prometaphases. Interestingly, side-on interactions in vector cells were characterized by being localized close to the end of the MT fiber, as the KT had already been moved toward the MT plus-end during the MG132 time (inset 3); in EGFP-Hec1 prometaphases a large part of side-on interactions were lateral interactions with the MT wall on a continuing MT (inset 6). This further suggests that initial side-on attachments in EGFP-Hec1 cells persist during prometaphase, as they were more stable than side-on attachments in normal prometaphase cells. We then assayed the effects of these persisting EGFP-Hec1-induced interactions on K-fiber stability. Hepatoma Up-Regulated Protein (HURP) is a MT associated protein that is involved in K-fiber stabilization at KT [43]–[45]. Given this specific HURP function, we analyzed HURP localization upon EGFP-Hec1 expression. In a normal mitotic cell HURP localized predominantly to the MT fiber anchored at the KT in the vicinity of chromosomes, (Figure 5B, vector). In contrast, HURP signal was spread along the K-fiber in EGFP-Hec1 cells (Figure 5B, EGFP-Hec1, upper row), and reached the centrosomal area in some mitoses (Figure 5B, EGFP-Hec1, lower row), suggesting that K-fibers were stabilized in EGFP-Hec1 cells. To assess the overall stability of MTs in these conditions, HeLa cells were shortly incubated with NOC and spindle MTs were visualized by α-tubulin immunostaining. Most vector-transfected mitotic cells showed totally depolymerized spindle MTs or exhibited rare K-MTs (Figure 5C). On the contrary, a high fraction of mitoses possessed NOC-resistant MTs among EGFP-Hec1 cells with MTs remaining partly polymerized (Figure 5C). Interestingly, analysis of the residual MT bundles in relation to KT position showed that stabilized MTs were connected to a KT or localized in the middle of a group of KTs (Figure 5C, inset), suggesting that stabilization of the MT fiber in EGFP-Hec1 cells occurred predominantly at KT. Taken together, these results indicate that K-fibers are stabilized in the presence of EGFP-Hec1 induced mis-attachments.

### EGFP-Hec1 mis-attachments allow partial MT dynamics at kinetochore

**Figure 6:**
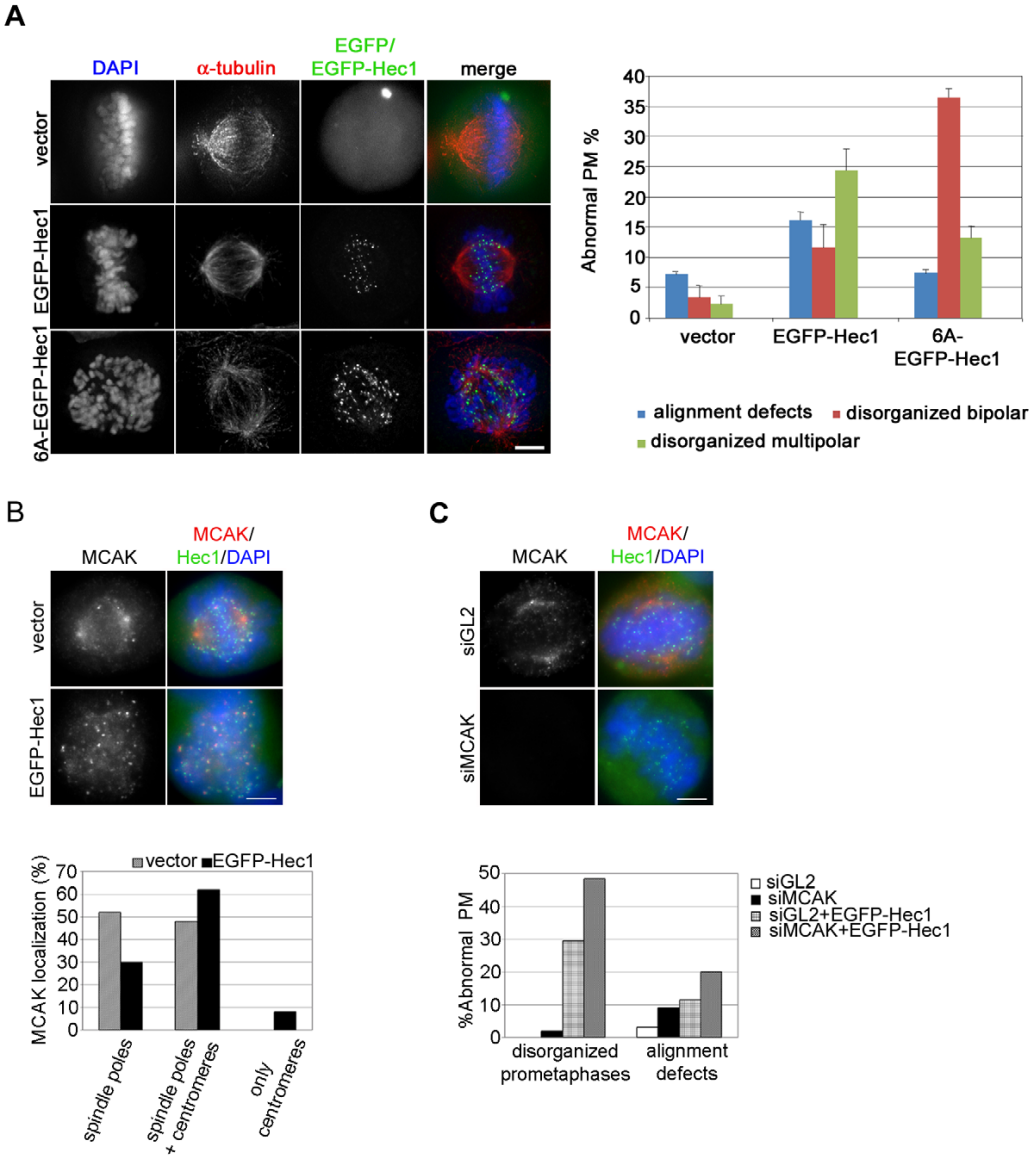
EGFP-Hec1 misattachments allow a partial MT dynamics at kinetochore. (A) EGFP, EGFP-Hec1 or 6A-EGFP-Hec1 (green) expressing cells stained for α-tubulin (red) and DAPI (blue). The vast majority of chromosomes were unaligned in the 6A-EGFP-Hec1 prometaphase. The graph shows a quantitative analysis of prometaphase abnormalities in EGFP (vector), EGFP-Hec1 or 6A-EGFP-Hec1 expressing cells. (B) EGFP and EGFP-Hec1 cells were stained with antibodies to MCAK (red) and Hec1 (only for vector, green), DNA in blue. MCAK accumulated on several centromeres in the EGFP-Hec1 prometaphase. The graph shows a quantitative analysis of MACK localization in mitotic cells (n = 50). (C) MCAK (red) staining after control (GL2)- or MCAK-siRNA transfection in EGFP-Hec1 (green) cells. MCAK was efficiently depleted in the EGFP-Hec1 cell. The graph shows the percentage of abnormal phenotypes in GL2- or MCAK-silenced prometaphase/metaphase (PM/M) cells (n≥100) with or without EGFP-Hec1 expression, as scored by MCAK and DAPI staining; Both alignment defects (P<0.05, χ^2^ test) and disorganized prometaphases (P<0.01, χ^2^ test) were higher in MCAK-siRNA/EGFP-Hec1 than in GL2-siRNA/EGFP-Hec1 cells. Bars, 5 µm.

It is well known that the N-terminal region of Hec1 is phosphorylated *in vitro* and *in viv*o by Aurora B kinase [24], [26], [28] and that its phosphorylation status regulates plus-end MT attachment stability at the KT. In line with this, PtK-1 cells expressing a mutant Hec1 plasmid in which six Aurora B target residues were mutated to alanine (6A-EGFP-Hec1) to prevent Aurora B phosphorylation undergo an extremely impaired chromosome congression with more than half of mitoses exhibiting mostly unaligned chromosomes and an increase in merotelic attachments as a consequence of strong stabilized KT-MT attachments [24]. Since our microscopic analysis identified calcium resistant side-on attachments in EGFP-Hec1 expressing cells, we assessed whether this phenotype was dependent on inhibition of Aurora B-mediated phosphorylation of N-terminally tagged Hec1. Cells transiently expressing 6A-EGFP-Hec1 were analyzed and compared to cells in which the six phosphorylatable residues were present (Figure 6A). As expected, EGFP-Hec1 expression induced both disorganized bipolar prometaphases and disorganized multipolar prometaphases (Figure 6A, graph). On the contrary, most 6A-EGFP-Hec1 mitoses were disorganized bipolar promataphases which displayed profound alignment defects with most chromosomes unaligned with respect to the spindle equator (Figure 6A, 6A-EGFP-Hec1). Indeed, spindle multipolarity was reduced in non-phosphorylatable cells (Figure 6A, graph), consistent with our time lapse results showing that spindle fragmentation occurs after chromosomes try to congress to the metaphase plate (Figure 4C). These data are in accordance with the finding that lack of Aurora B mediated phosphorylation on Hec1 N-terminal tail produces hyperstable KT-MT interactions that dramatically impair chromosome congression leaving most chromosomes monooriented [24]. They also show that defective KT-MT attachments induced by the presence of the EGFP tag still allow a residual dynamics on MT plus-ends which permits a partial congression toward the center of the spindle, as a consequence of the interaction of both sister kinetochores with spindle microtubules.

Mitotic Centromere-Associated Kinesin (MCAK) controls MT dynamics at MT plus-end [46], [47] and is thought to participate in the correction pathway for erroneous KT-MT interactions by promoting MT turnover at KT [48], [49]. In light of this, we decided to investigate whether MCAK localization was altered in EGFP-Hec1 cells. In most vector-transfected cells MCAK localized either to spindle poles only (not shown) or to spindle poles and a few KTs (Figure 6B, vector). In EGFP-Hec1 expressing cells more mitoses showed MCAK both at poles and KTs and several EGFP-Hec1 cells, but no vector transfected cell, showed MCAK only at KTs (Figure 6B, EGFP-Hec1 and graph). Furthermore, a higher number of centromeres were positive for the MCAK signal in each cell (38% mitoses with >5 positive centromeres in EGFP-Hec1 *vs* 10% in vector mitoses, n = 50). These results clearly show that MCAK re-localizes from centrosomes to centromeres upon EGFP-Hec1 expression, in line with previous reports showing that erroneous KT-MT interactions *i.e.* merotelic attachments recruit MCAK at KTs [49]. To further assess whether MCAK re-localization in EGFP-Hec1 cells was connected to its function in the correction of faulty KT-MT attachments into functional end-on attachments, we induced siRNA-mediated depletion of MCAK in the cells that expressed EGFP-Hec1 (Figure 6C). In MCAK-depleted/EGFP-Hec1 expressing cells the frequency of abnormal prometaphases significantly increased in comparison with control GL2-depleted/EGFP-Hec1 expressing cells (Figure 6C, graph). Thus, this set of data together with the data showing K-fiber stabilization and defective correction of congression defects in EGFP-cells suggest that side-on KT-MT attachments generated by EGFP-Hec1 containing Ndc80 complexes are subjected to an altered dynamics in which MCAK-dependent MT turnover at KT is inefficient.

### Impairing effect of EGFP-Hec1 is mediated by the motor activity of CENP-E

**Figure 7:**
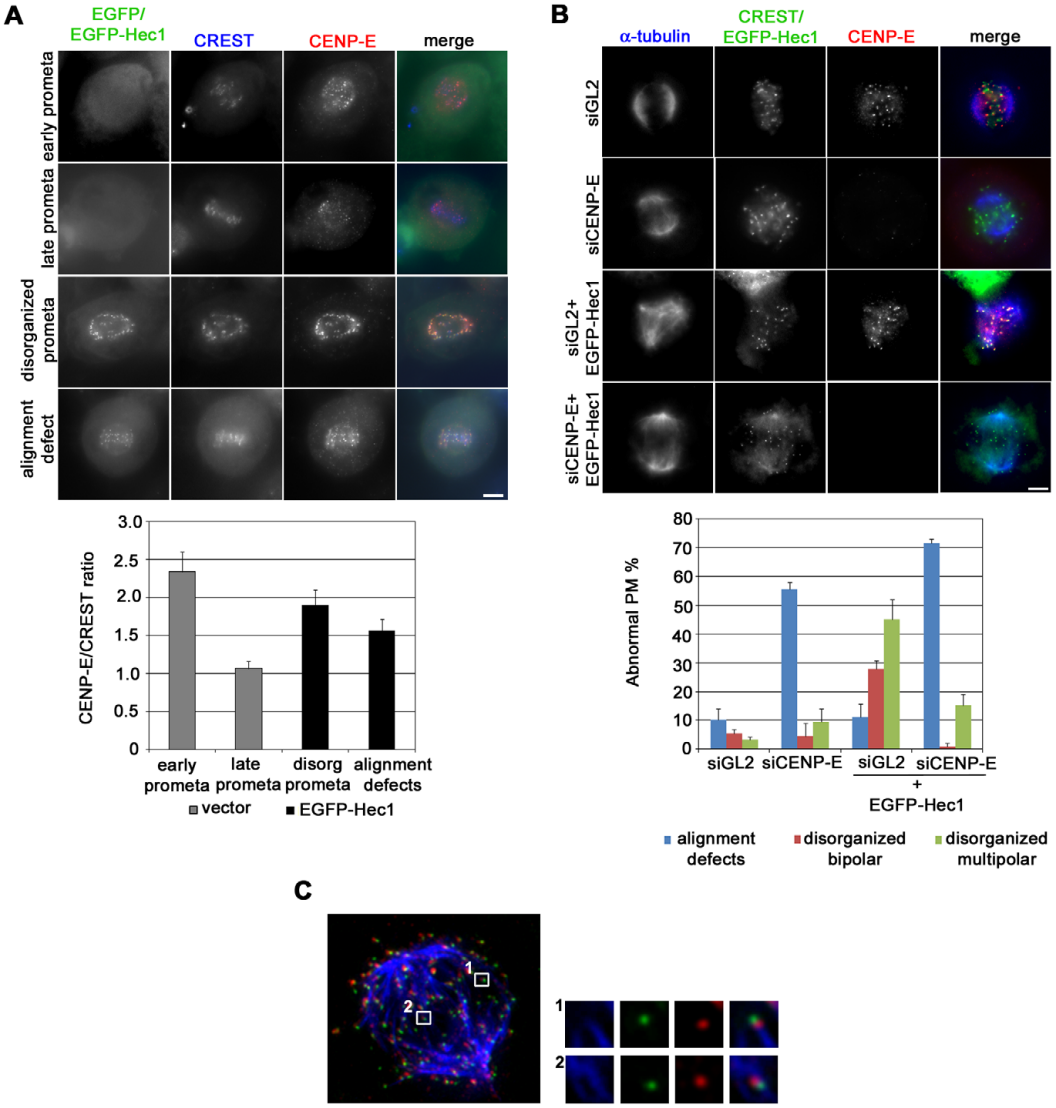
CENP-E function is implicated in centrosomal fragmentation. (A) Kinetochore accumulation of CENP-E (red) in an early and a late normal prometaphase from vector transfected cultures and in a disorganized prometaphase and a late prometaphase with alignment defects from EGFP-Hec1 (green) trasfected cultures. Kinetochores are marked by CREST staining (blue). The graph shows a quantitative analysis of CENP-E intensity at kinetochores, in vector and EGFP-Hec1 transfected cells. CENP-E/CREST ratio was significantly higher in disorganized prometaphases (P<0.01, t-test) and in prometaphases with alignment defects (P<0.05) from EGFP-Hec1 expressing cells than in normal late prometaphases from vector transfected cells. (B) Chromosome congression and spindle organization in control (siGL2) or CENP-E silenced (siCENP-E) mitoses or mitoses expressing EGFP-Hec1 after control silencing (siGL2+ EGFP-Hec1) or CENP-E silencing (siCENP-E + EGFP-Hec1). Mitoses were observed after α-tubulin (blue) and CENP-E (red) staining. Kinetochores were identified by CREST staining in siGL2 and siCENP-E samples or by EGFP expression in induced samples (green). The graph shows the frequencies of abnormal PM in the different conditions. Data are mean ± SE of >150 PM scored for each condition in two experiments. Bars, 5 µm. (C) KT-MT attachments in an EGFP-Hec1 (green) expressing disorganized bipolar prometaphase exposed to MG132 for 2 h and stained for CENP-E (red) and α-tubulin (blue) after calcium buffer treatment. A maximum projection from deconvolved z-stacks is shown. Insets show a 3-fold enlargement of one optical slice from the boxed regions. An end-on attachment (inset 1) and a side-on attachment (inset 2) are shown.

We then moved to search for potential candidates for the generation of forces promoting pole fragmentation and centriole splitting and analyzed force-producing kinetochore motors [50]. Thus, we investigated the mitotic behavior of dynein, a minus end directed molecular motor, but we found that it correctly accumulated on KTs of polar chromosomes and then delocalized from KTs of aligned chromosomes in EGFP-Hec1 expressing cells (not shown). We next analyzed CENP-E, a plus-end directed motor protein that stabilizes initial KT-MT interactions and has been reported to directly interact with Nuf2, the Hec1 sub-complex partner [51], [52], [53]. During mitosis, CENP-E highly accumulates on prometaphase KTs, then partially delocalizes from KTs when end-on attachments are formed, and it is not visible anymore on anaphase KTs [51], [52]. Accordingly, CENP-E antibody intensity at KT was definitively lower in late prometaphases/metaphases relative to early prometaphases in vector-transfected cultures (Figure 7A). On the contrary, EGFP-Hec1 KTs both in prometaphases with alignment defects and in disorganized prometaphases accumulated CENP-E at levels that were intermediate between those of early and late prometaphases (Figure 7A), suggesting that persisting initial interactions driven by the EGFP-Hec1 protein retained CENP-E at KTs. We then depleted CENP-E from EGFP-Hec1 expressing cells to assess whether the KT-directed forces produced by this motor protein were required for pole fragmentation. As expected, CENP-E silencing produced defective chromosome alignment with numerous chromosomes located in the polar region (Figure 7B), in accordance with its role in chromosome congression [52], [54]. In CENP-E depleted samples expressing the chimeric Hec1 protein the frequencies of disorganized prometaphases showing multipolar or bipolar spindles dropped dramatically and a vast majority of cells showed many unaligned chromosomes, similarly to CENP-E depleted cells (Figure 7B). To further define the role of CENP-E in mediating force generation on EGFP-Hec1 kinetochores, we visualized the kinesin on MT–KT interactions after EGFP-Hec1 induction (Figure 7C). Indeed, we found that EGFP-Hec1 mediated lateral attachments clearly recruited high levels of CENP-E on kinetochores localizing in the central region of the spindle (inset 2 in Figure 7C). Collectively, these data strengthen the conclusion that spindle pole fragmentation is mediated by forces exerted on K-fibers and identify the plus-end directed kinesin CENP-E as a force-generating motor protein at EGFP-Hec1 KTs.

## Discussion

The present work shows that expression of a N-terminally modified Hec1 induces mitotic aberrations in human cells, namely defective chromosome congression and formation of multipolar spindles as a consequence of centriole splitting. Analysis of the mitotic process in EGFP-Hec1 expressing cells demonstrates that Ndc80 complexes harboring EGFP-tagged Hec1 localize at kinetochores but the N-terminally tagged protein interferes with the Ndc80 dependent MT dynamics during the pre-anaphase stages of mitosis. Our findings provide evidence that mitotic aberrations are the consequence of improper KT-MT interactions, including persisting attachments between the KT and the MT lateral wall. According to *in vitro* reconstitution experiments of the Ndc80 complex, the Hec1 subunit of the Hec1/Nuf2 sub-complex interacts with MTs along the MT lattice [26]–[28], a feature that could promote the formation of lateral attachments during the initial stages of chromosome congression in vertebrate cells [55]. Our data indicate that the dynamic rearrangements of KT-MT interactions needed to transform lateral interactions into functional end-on attachments are impaired by the presence of the EGFP tag at the N-terminus of Hec1. A collected body of *in vitro* and *in vivo* evidence indicates that the highly basic 80 aminoacid N-terminal tail domain of Hec1 is required for the interaction between the Ndc80 complex and MT by its direct binding to the C-terminal acidic tail domain of tubulin exposed on the MT surface [28], [56], [57]. These findings provide an explanation for the impairing effect of EGFP-Hec1 on KT-MT interaction dynamics, since the presence of several negatively charged patches on the barrel shaped structure of the EGFP [58] might enhance electrostatic interactions between the Hec1 tail and MTs [59], interfering with the conformational changes of the 80 aminoacid tail required for KT-MT attachment dynamics during congression [56], [57]. It has been reported that Aurora B dependent phosphorylation of residues within the 80 aminoacid tail domain of Hec1 regulates MT attachment stability in higher eukaryotes [24], [27], [28]. Concordantly, mutation of multiple residues in the tail domain preventing Aurora B phosphorylation *in vivo* produced robust kinetochore MT attachments, whereas mutation in these residues mimicking constitutive phosphorylation resulted in loss of interaction [24], [57]. In our data, comparison of the phenotypes due to phoshorylatable and non-phosphorylatable N-terminally tagged Hec1 in human cells demonstrates that the presence of EGFP still allows a residual MT plus-end dynamics and a partial prometaphase chromosome movement, as compared with the extreme phenotype produced by the lack of Aurora B phosphorylation. This indicates that EGFP presence at Hec1 N-terminal tail in EGFP-Hec1 cells does not impede Aurora B phosphorylation. Altogether, these data suggest that the influence of modified Hec1 on KT-MT attachment stability may be largely mediated by changes in electrostatic affinity between the Hec1 N-terminal tail and the C-terminal tubulin tails.

**Figure 8:**
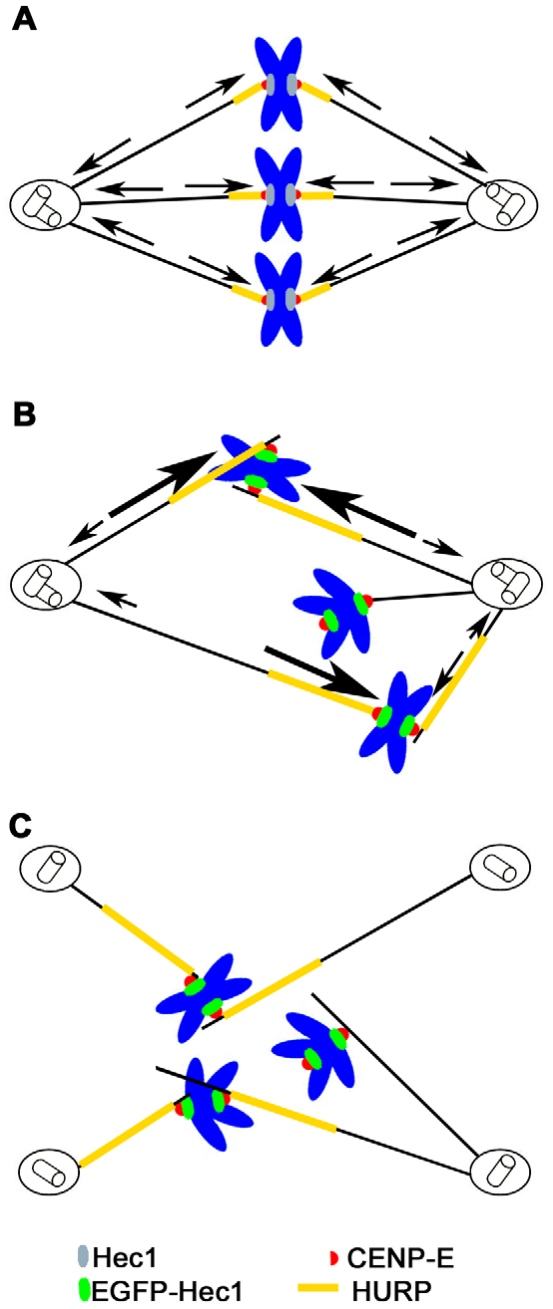
Schematic representation of the mechanism producing centrosome fragmentation after EGFP-Hec1 expression. (A) Metaphase cell with balanced spindle forces (arrows). (B) EGFP-Hec1 expressing prometaphase cell. EGFP-Hec1 kinetochores (green) form lateral kinetochore-microtubule attachments that recruit more CENP-E (red) and HURP (yellow). CENP-E mediated plus-end directed forces (large arrows) overcomes the dynein-mediated minus-end forces (small arrows). (C) Kinetochore directed CENP-E mediated forces produce centrosome fragmentation and centriole splitting.

A striking feature of EGFP-Hec1 expression was the induction of multipolar spindles caused by centriole splitting. Establishment and maintenance of a bipolar spindle is achieved through MT polymerization/depolymerization dynamics and the combined actions of minus-end and plus-end directed forces exerted by molecular motors. These forces are resisted by the intrinsic elasticity of subcellular structures, such as the centromere, as well as the cell inherent molecular friction [38]. All these functions result in a complex balance of pulling and pushing forces generated at spindle poles, along chromosome arms and at kinetochores that regulate spindle length and bipolarity [38]. Indeed, maintenance of a bipolar spindle has been shown to depend on the balance of opposing forces generated at spindle poles and kinetochores by the MT depolymerazing activity of the Kin 13 family members, Kif2a and MCAK, respectively [60]. Furthermore, spindle pole focusing associated to HSET and NuMA-dependent motor activities has been shown to be counteracted by forces generated at KT on K-fibers [61]. In our experiments, centrosome fragmentation and centriole splitting could be efficiently rescued by drugs affecting MT dynamics or antiparallel MT sliding, suggesting that EGFP-Hec1 at KTs is responsible for an unbalance in MT-mediated forces, which ultimately cause centrosome disruption. We also found that EGFP-Hec1-induced improper interactions stabilize K fibers by recruiting the MT-associated protein HURP. Consistent with this, recent work has shown that the mitotic spindle is heavily stabilized and MT dynamics is impaired when HURP localization is spread along the entire metaphase spindle, as after expression of the HURP-MT-binding domain [62]. Finally, the plus-end kinesin CENP-E was specifically retained to KTs after EGFP-Hec1 expression. We schematically propose a sequence of events that may explain the observed phenotypes in EGFP-Hec1 expressing mitoses (Figure 8). In a normal metaphase cell spindle bipolarity is maintained by the balance of plus-end and minus-end directed forces generated at end-on attached kinetochores and at centrosomes (Figure 8A). We hypothesize that kinetochores harboring a modified Ndc80 complex with EGFP N-terminally tagged Hec1 form abnormal lateral attachments to MTs. These lateral attachments retain CENP-E on KTs and recruit stabilizing factor on K-fibers, as a consequence of an impaired MT dynamics. This results in a defective congression of a large part of the chromosomes, in which both sister kinetochores are engaged in persisting MT lateral interactions (Figure 8B). When a substantial number of chromosomes are engaged in these faulty interactions on both sister kinetochores, plus-end directed CENP-E-dependent forces exerted on stabilized lateral KT-MT attachments produce a net pulling force on centrosomes which disrupts the innermost centrosomal structure and generate multipolar spindles (Figure 8C). In conclusion, altered KT-MT attachment stability may promote enhanced CENP-E-dependent force generation at kinetochore, disrupting the fine balance of forces regulating spindle formation and bipolarity. At present we cannot exclude that a defective interaction of EGFP-tagged Hec1 with the centrosomal microtubule-associated protein Hice1 may influence centrosome stability in EGFP-Hec1 mitoses, although we retain it unlikely since the Hec1 binding domain for Hice1 has been identified on its C-terminal region [63].

Interestingly, all these mitotic dysfunctions are brought about by the capacity of EGFP-Hec1 to accumulate at the expense of endogenous Hec1, thus forming chimeric complexes. We do not know at the moment the reason for the preferential accumulation of EGFP-Hec1 in comparison with untagged or C-tagged protein, although several hypotheses can be drawn, including differential protein stability or increased transcription due to tag localization. The fact that neither untagged Hec1 nor C-terminally tagged Hec1 are found to accumulate in cells indicates that intracellular Hec1 levels are finely regulated, possibly by activating degradation of Hec1 molecules not engaged in protein interactions with Nuf2. These cellular buffering mechanisms are disrupted in our single site integration transfection strategy only when a N-terminally tagged Hec1 is expressed. In this context, inducible Hec1 overexpression observed in a mouse system [35] could be explained by the tag localization at the N-terminal tail of Hec1 or by differences in the expression strategy used in that study as compared to the present work.

Altogether, our work indicates that modifications on the Hec1 N-terminal tail may alter KT-MT attachment stability and influence Ndc80 complex function independently from the intracellular levels of the protein. It will be interesting to investigate whether mutations or post-translational modifications of this Hec1 region are characteristic of cancer cells. Furthermore, expression of N-terminal modified Hec1 in cancer cells can be used to specifically induce chromosome segregation within multipolar spindles and trigger the formation of extremely aneuploid and/or polyploidy daughter cells that will be effectively eliminated from the growing population (Totta and Degrassi, unpublished). In line with this, induction of massive aneuploidy has been already suggested as a strategy to efficiently kill cancer cells [9]. In conclusion, the findings presented in this paper provide a novel mechanism for the generation of mitotic failure, *i.e.* enhanced CENP-E generated kinetochore-directed forces that are able to disrupt spindle integrity. It will be interesting to further understand the role of depolymerases or microtubule associated proteins present on the kinetochore [64] and interacting with CENP-E at kinetochore [65] in this process. Our findings support a model in which spindle architecture is governed by integrated activities operating at centrosomes and kinetochores, so that centrosome integrity is influenced by the pathways regulating kinetochore-microtubule attachment stability.

## Materials and Methods

### Cell culture, transfection, RNAi and drug treatment

HeLa cells were grown in DMEM supplemented with 10% foetal bovine serum (both from Euroclone), 1% L-Glutamine and antibiotics at 37°C. Full-length *HEC1* cDNA (accession NM_006102) was amplified by PCR and cloned into the pEGFP-C1 vector (BD Biosciences Clontech). DNA sequence of the recombinant plasmid was verified. Doxy-inducible stable cell lines were created as previously described [36]. Briefly, the pEGFP-C1-HEC1 insert was mutagenized to create a siRNA-resistant allele, cloned into pcDNA5/FRT/TO plasmid (Invitrogen) and modified to contain an N- or C-terminal EGFP tag. Vectors were then co-transfected into Flp-In TReX tetracycline transactivator HeLa cells (Invitrogen) with the Flp recombinase encoding plasmid pOG44 (a kind gift from Dr Taylor). Hygromycin-resistant single cell clones were picked and expanded to obtain clonal cell lines. Transgene expression was induced by the addition of 20–200 ng/ml doxy (Sigma-Aldrich) for 24 h or 36 h. In silencing experiments 2×10^5^ cells were plated in 35 mm Ø Petri dishes the day before RNAi. 20 nM siRNA targeting HEC1 (5′-AAGTTCAAAAGCTGGATGATC-3′) [18], CENP-E (5′-AAACACTTACTGCTCTCCAGT-3′) [66], or control siRNA against firefly luciferase (GL2) sequence (5′-CGTACGCGGAATACT TCGA-3′) [67] were supplied to cells using Lipofectamine 2000 (Invitrogen) according to the manufacturer's instructions. 24 h later doxy was added for further 24 h. To transiently transfect cells, 2×10^5^ HeLa cells were plated in 35 mm Ø Petri dishes the day before transfection. The next day 0.5 µg of EGFP-Hec1 or pEGFP plasmid were transfected onto each dish as described above. In silencing experiments on HeLa cells 60 nM siRNA targeting MCAK (5′-GATCCAACGCAGTAATGGT-3′) [68] were added to the EGFP-Hec1 transfection mixture and transfected onto the Petri dishes as described. Cells were assayed 48 h post-transfection. To analyse spindle pole fragmentation, EGFP-Hec1 or empty vector transfected cells were incubated with 1 µM NOC, 15 nM TAX or 100 µM MON (all from Sigma) for the last 4 h before harvesting. To assay MT stability cells were incubated 10 min at 37°C with 1 µg/ml NOC. KT-MT interactions were analyzed in EGFP-Hec1 expressing cells incubated for the last 2 h in growth medium with 10 µM MG132 (Chemicon).

### Immunoblotting

Cells were lysed for 30 min on ice in RIPA buffer (50 mM Tris, 150 mM NaCl, 1 mM EGTA, 1 mM EDTA, 1 mM sodium deoxycholate, 1% NP40) supplemented with protease inhibitors (all from Sigma). After determination of protein concentration (Bradford reagent, Sigma), 40 µg of total proteins were resolved in SDS-PAGE with NuPAGE® Bis-Tris precasted 10% or 4-12% gels, run in NuPAGE® MOPS buffer (Invitrogen) under reducing conditions and transferred onto nitrocellulose membranes (Schleicher and Schuell). Membranes were blocked in blocking buffer (5% skim milk in TBS +0.1% Tween 20) and incubated with mouse anti-Hec1 (9G3; AbCam, 1∶1000), goat anti-actin (Santa Cruz, 1∶400) or mouse anti-vinculin (Sigma) antibodies in blocking buffer. Goat anti-mouse HRP (Chemicon, 1∶5000) or donkey anti-goat HRP antibody (Santa Cruz, 1∶5000) were successively applied. Antigens on the membrane were revealed by enhanced chemiluminescence (ECL plus, Amersham). Densitometric analysis of immunoblots was performed using ImageJ (http://imagej.nih.gov/ij/) software.

### Immunofluorescence

Cells were rinsed in PHEM (60 mM PIPES, 25 mM Hepes, 10 mM EGTA, 2 mM MgCl_2_) fixed for 15 min with 3.7% formaldehyde in PHEM, permeabilized 5 min in 0.3% Triton X-100 and post-fixed in cold methanol for 3 min. For centrin or CENP-E staining, cells were fixed 6 min in ice-cold methanol. To assess MT stability cells were treated with MT buffer (100 mM PIPES, 1 mM MgCl_2_, 0.1 mM CaCl_2_, 0.1% Triton X-100) for 90 sec and then fixed for 15 min in 3.7% formaldehyde in MT buffer. Coverslips were blocked in PBS containing 20% goat serum (GS) for 30 min at 37°C, before being processed for immunofluorescence. Antibody dilution was as follows: human anti-KT serum (CREST; Antibodies Inc., 1∶40); mouse α-tubulin (DM1α; Sigma, 1∶400); rabbit γ-tubulin (Sigma, 1∶1000); mouse centrin (kindly provided by J. Salisbury, 1∶400); rabbit HURP (kindly provided by E. Nigg, 1∶300); mouse Hec1 (9G3; AbCam, 1∶1000); FITC-anti-α-tubulin (Sigma, 1∶100); rabbit CENP-E (Santa Cruz, 1∶200); rabbit Spc24/25 ([29], 1∶1000). Secondary antibodies conjugated to Alexa-488 (Molecular Probes), Rhodamine-RedX or AMCA (Jackson Laboratories) were chosen as appropriate and used as recommended by the supplier. For MCAK detection, cells were fixed with 1% formaldehyde in PHEM and permeabilized in 0.5% Triton X-100, then blocked in PBS +20% GS and processed for indirect labelling with 0.5 µg/ml of a rabbit anti-MCAK antibody (kindly provided by C. Walczak) and a RedX-secondary antibody. Where indicated, DNA was counterstained with 0.1 µg/ml 4′-6-Diamidino-2-phenylindole (DAPI, Sigma). Coverslips were sealed in antifade solution (Vector). Preparations were examined under an Olympus AX70 microscope using a 100×/1.35NA objective. Images were acquired using a SPOT charge-coupled device camera (Diagnostic Instruments) controlled by ISO 2000 software (DeltaSistemi) and processed using either Adobe Photoshop 7.0 or NIH ImageJ 1.3 software. For KT-MT interaction analysis, z-stacks of optical sections were acquired at 0.3 µm intervals and images were deconvolved and reconstructed as maximum projections using AutoDeblur 9.3 software (AutoQuant Imaging Inc.). Measurements of fluorescence intensity were obtained from single optical sections of images acquired under identical exposure settings.

### Live imaging

Mitotic progression of HeLa cells cotransfected with EGFP-Hec1 and mCherry-human-α-tubulin (a kind gift of R. Tsien) vectors was followed under a Leica SP5 confocal microscope equipped with a 37°C chamber and a 63X oil objective. Data were processed with NIH ImageJ 1.3 software.

## Supporting Information

Figure S1 Doxycycline**-**inducible expression of differently tagged Hec1. Western blot analysis of Hec1-EGFP (A) or EGFP-Hec1 (B) after several doxy doses.
(TIF)

Figure S2 Kinetochore localization of Ndc80 modified complexes. Hec1-EGFP (A) or EGFP-Hec1 (B) localization in prometaphase cells stained for the Ndc80 complex subunits Spc24/Spc25 (red) and DAPI (blue). Maximum projections from deconvolved Z-stacks are shown. Bar, 5 µm.
(TIF)

Figure S3 Transient EGFP-Hec1 expression disrupts mitosis in HeLa cells. (A) Hec1 expression after flow sorting of EGFP-Hec1 positive and negative cells. (B, C) Chromosome congression defects in EGFP or EGFP-Hec1 (green) transfected cells stained for CREST (red) and DAPI (blue). Data are the mean ± SE of 4 independent experiments, >400 cells scored for each experimental point. Disorganized prometaphases and alignment defects (as assessed by CREST and DAPI staining) were significantly higher in EGFP-Hec1 mitoses (P<0.01, t-test). (D) Duration of the different stages of mitosis in EGFP-Hec1 and control cells. (E) Selected phase contrast frames of an EGFP-Hec1 mitotic cell imaged by time-lapse microscopy. The cell undergoes anaphase with chromatin lying behind and fails cytokinesis. Arrows point to chromatin remaining at the equator of the spindle. Time in min; Bar, 5 µm.
(TIF)

Figure S4 Abnormal phenotypes and spindle defects after transient EGFP-Hec1 expression in different cell lines. (A, C) Chromosome congression defects in vector and EGFP-Hec1 transfected U2OS (A) or HCT116 (C) cells stained for CREST and DAPI. (B, D) Spindle multipolarity as assessed by α-tubulin staining in vector and EGFP-Hec1 transfected U2OS (B) or HCT116 (D) cells.
(TIF)
