# Supplementary figures and images for: Abnormal Kinetochore-Generated Pulling Forces from Expressing a N-Terminally Modified Hec1
Source: PLoS One. 2011 Jan 28;6(1):e16307. doi: 10.1371/journal.pone.0016307 (PMC3030568; doi:10.1371/journal.pone.0016307)

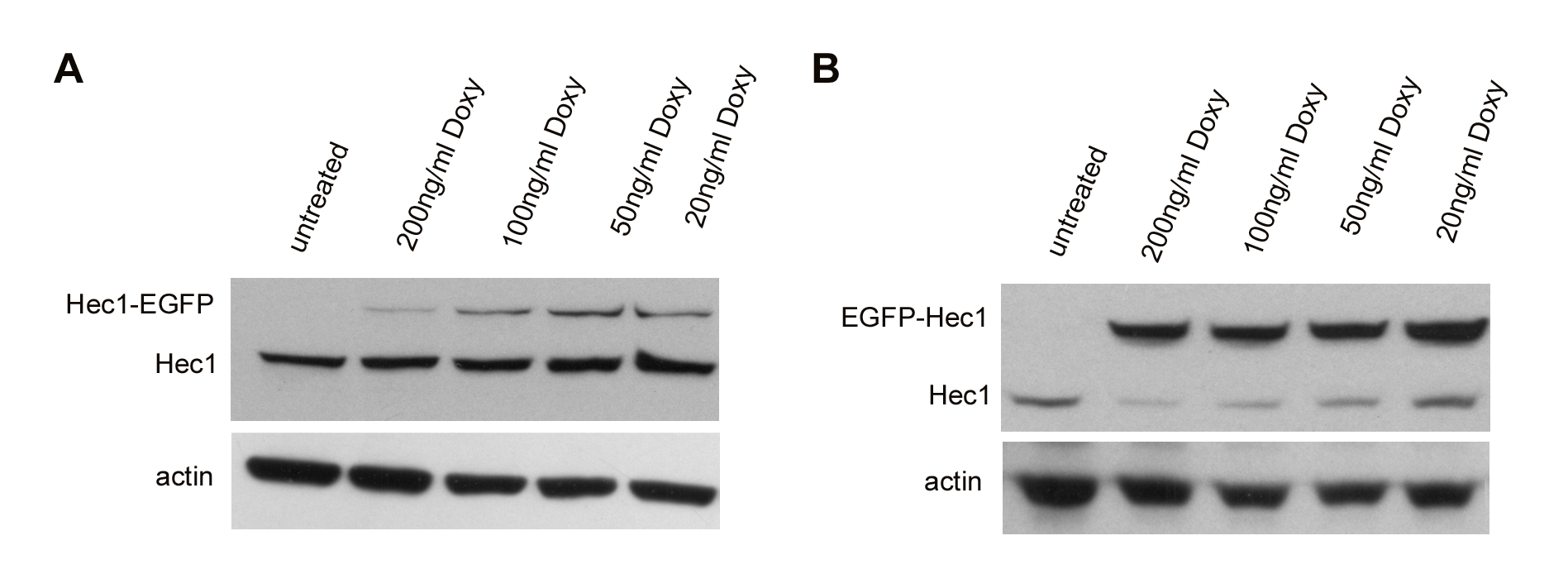

Supplement: Figure S1 — Doxycycline-inducible expression of differently tagged Hec1. Western blot analysis of Hec1-EGFP (A) or EGFP-Hec1 (B) after several doxy doses. (TIF) [file pone.0016307.s001.tif]

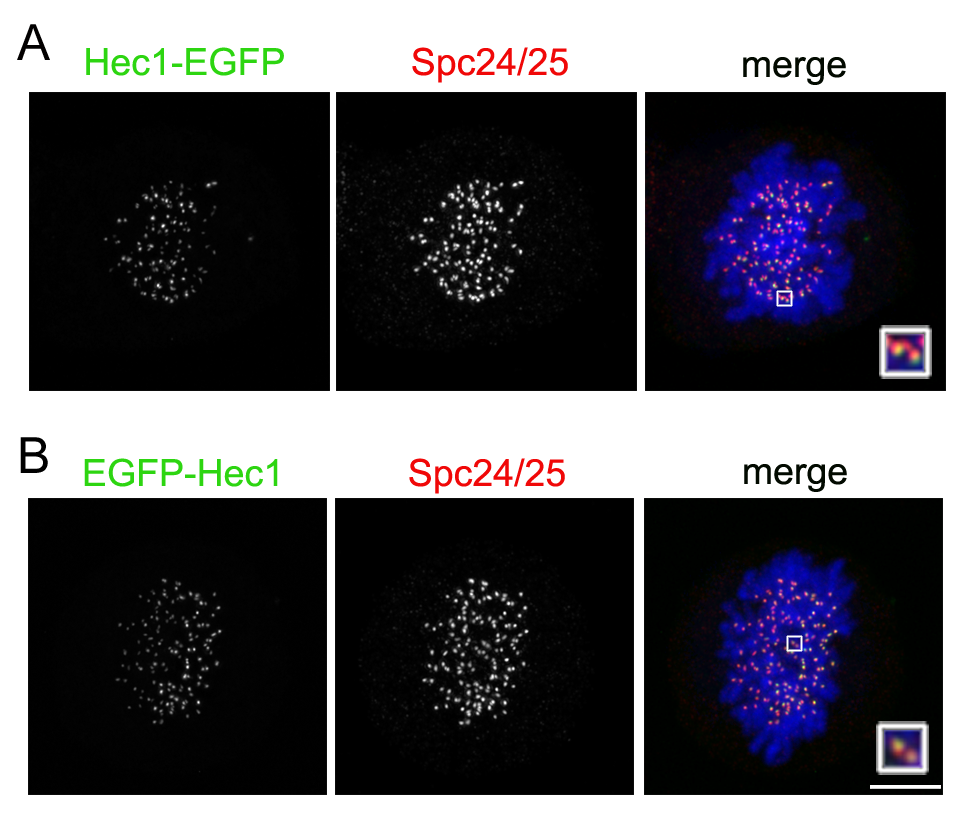

Supplement: Figure S2 — Kinetochore localization of Ndc80 modified complexes. Hec1-EGFP (A) or EGFP-Hec1 (B) localization in prometaphase cells stained for the Ndc80 complex subunits Spc24/Spc25 (red) and DAPI (blue). Maximum projections from deconvolved Z-stacks are shown. Bar, 5 µm. (TIF) [file pone.0016307.s002.tif]

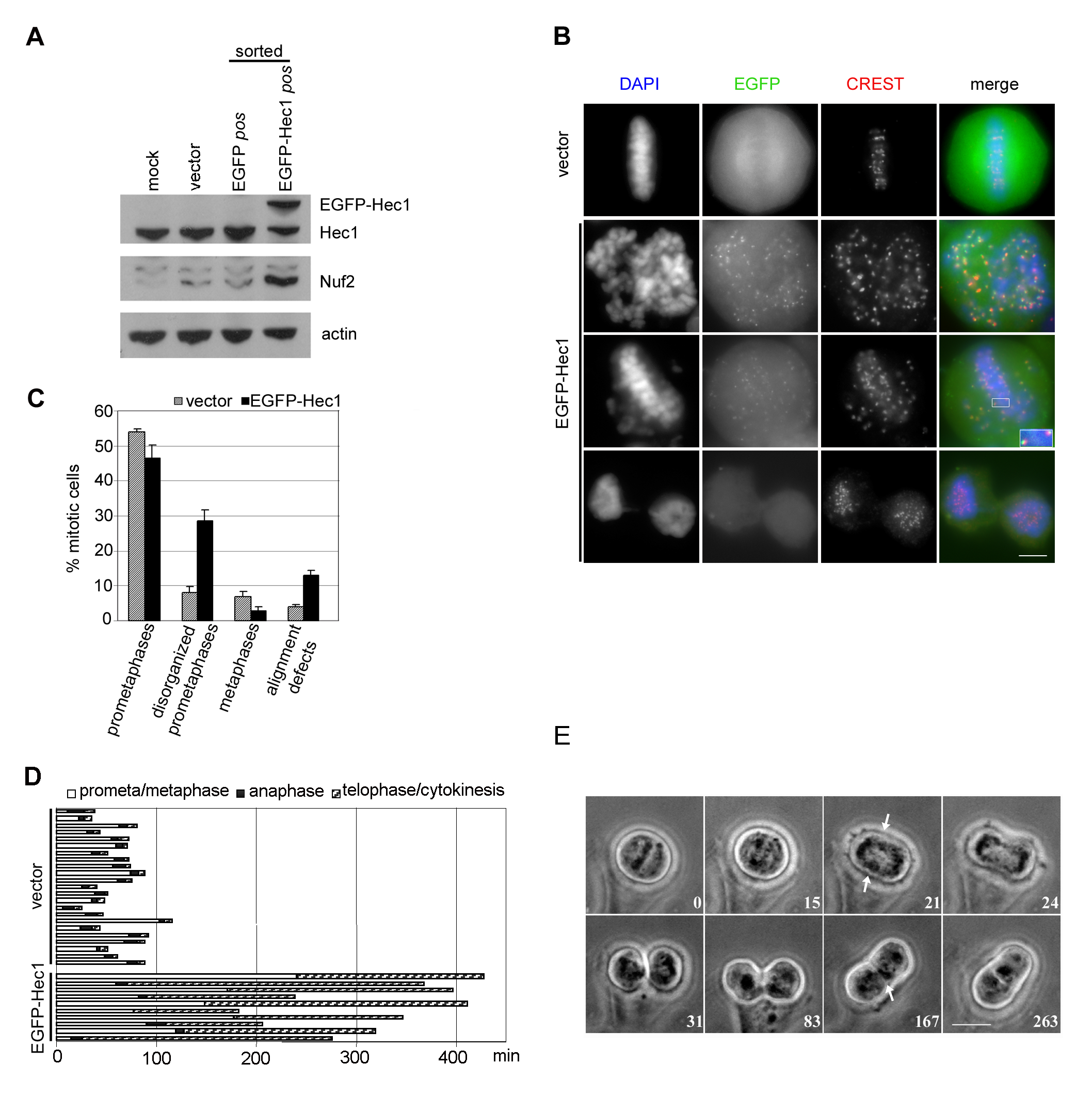

Supplement: Figure S3 — Transient EGFP-Hec1 expression disrupts mitosis in HeLa cells. (A) Hec1 expression after flow sorting of EGFP-Hec1 positive and negative cells. (B, C) Chromosome congression defects in EGFP or EGFP-Hec1 (green) transfected cells stained for CREST (red) and DAPI (blue). Data are the mean ± SE of 4 independent experiments, >400 cells scored for each experimental point. Disorganized prometaphases and alignment defects (as assessed by CREST and DAPI staining) were significantly higher in EGFP-Hec1 mitoses (P<0.01, t-test). (D) Duration of the different stages of mitosis in EGFP-Hec1 and control cells. (E) Selected phase contrast frames of an EGFP-Hec1 mitotic cell imaged by time-lapse microscopy. The cell undergoes anaphase with chromatin lying behind and fails cytokinesis. Arrows point to chromatin remaining at the equator of the spindle. Time in min; Bar, 5 µm. (TIF) [file pone.0016307.s003.tif]

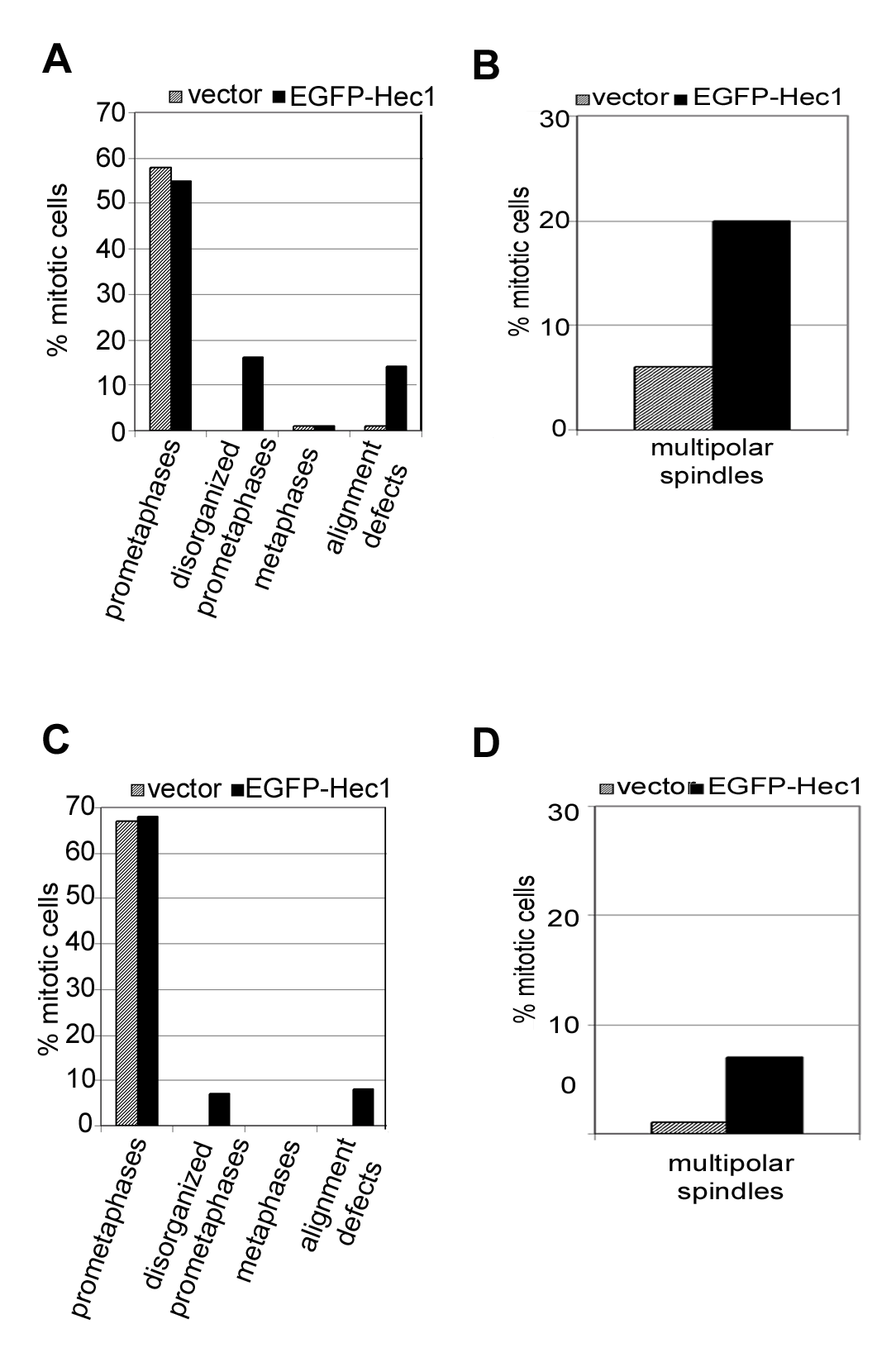

Supplement: Figure S4 — Abnormal phenotypes and spindle defects after transient EGFP-Hec1 expression in different cell lines. (A, C) Chromosome congression defects in vector and EGFP-Hec1 transfected U2OS (A) or HCT116 (C) cells stained for CREST and DAPI. (B, D) Spindle multipolarity as assessed by α-tubulin staining in vector and EGFP-Hec1 transfected U2OS (B) or HCT116 (D) cells. (TIF) [file pone.0016307.s004.tif]
